# “Vaccinating a child is upon the woman”: implications for improving uptake for the recently introduced second dose of measles-containing vaccine based on a rapid community assessment in Uganda

**DOI:** 10.3389/fgwh.2025.1441242

**Published:** 2025-04-11

**Authors:** Adelline Twimukye, Nessa Ryan, Flavia Vivian Najjuma, Yvette Wibabara, Judith Nanyondo, Shillah Nakato, Maria Sarah Nabaggala, Ciara Sugerman, Daniel Kadobera, Rita Atugonza, John Kamulegeya, Joseph Magoola, Racheal Beyagira, Mohammed Lamorde, Alex Riolexus Ario, Alfred Driwale, Shibani Kulkarni

**Affiliations:** ^1^ Infectious Diseases Institute, College of Health Sciences, Makerere University, Kampala, Uganda; ^2^ Global Immunization Division, US Centers for Disease Control and Prevention, Atlanta, GA, United States; ^3^ Global Immunization Division, US CDC, US Centers for Disease Control and Prevention, Kampala, Uganda; ^4^ Uganda National Institute of Public Health, Ministry of Health, Kampala, Uganda; ^5^ Vaccines and Immunization Division, Ministry of Health, Kampala, Uganda; ^6^ Programs Department, African Field Epidemiology Network, Kampala, Uganda

**Keywords:** caregiver barriers, social ecological, life course, measles vaccination, second year of life, demand, Uganda

## Abstract

**Background:**

Caregiver barriers to accessing immunizations are a key factor influencing childhood vaccination. In preparation for the rollout of the second dose measles-containing vaccine (MCV2) in Uganda in October 2022, we aimed to identify possible barriers specific to female caregivers that could influence MCV2 implementation and suggest initiatives to facilitate MCV2 uptake.

**Methods:**

In September 2022, we conducted a rapid community assessment in 18 districts in Uganda. We conducted key informant interviews with 17 district health managers and 18 community leaders, and 18 focus group discussions, one in each district, with caregivers of immunization-eligible children. We conducted a rapid analysis based of debriefing notes and in-depth thematic analysis of translated transcripts. Data were analyzed using NVivo version 12, wherein we used the framework analysis approach to define and structure codes deductively and inductively to identify themes. We mapped themes onto the socio-ecological model to examine factors that influence immunization at individual, household, community, and health system level.

**Results:**

We found that individual, household, and health system factors influenced childhood vaccination and could be potential barriers to MCV2 uptake. At the individual level, female caregiver's heavy workload and limited decision-making power hindered their ability to take children for vaccination, with mothers often relying on fathers and depended on men for transport costs to immunization centers. At the household level, participants mothers were primarily responsible for taking children to vaccination centers, while fathers were less involved in child health. Health workers often gave preferential treatment to fathers over mothers at the health facility when they brought the child in for vaccination Participants suggested that approaches that ensure the involvement of fathers, other family members and mother-to-mother peer groups could address the barriers specific to female caregivers.

**Conclusion:**

Role differentiation between female and male caregivers affect childhood vaccination practices within communities in Uganda, potentially exacerbating challenges in accessing vaccines for children in the second year of life. Integrating interventions responsive to specific caregiver needs and that improve family participation may improve childhood vaccination in Uganda.

## 1 Background

In 2009, the World Health Organization (WHO) recommended introducing the second dose of measles-containing vaccine (MCV2) in the routine immunization schedule once countries have achieved ≥80% coverage of MCV1 at the national level for 3 consecutive years (1). In 2017, this policy was revised to recommend that countries include MCV2 in their national vaccination schedules regardless of the level of MCV1 coverage (1). In Uganda, under-five mortality rates are high, with 9,774, significant measles cases reported, with mortality rates of 43 deaths per 1,000 live births. Introduction of MCV2 could improve vaccine coverage reduce outbreaks and enhance, the National Immunization Strategy 2022–2026 in Uganda. However, several challenges remain for its rollout in Uganda, as other African countries also struggle with low MCV2 coverage. The African region has shown slow progress in achieving optimal MCV2 coverage, with current coverage remaining sub-optimal in countries like Malawi (8%), Niger (16%), Angola (26%), Kenya (28%), Zambia (47%), and Burkina Faso (50%) (1).

Childhood vaccination coverage is influenced through multiple factors, such as demand, access, and uptake and quality of services received (2–5). There are culturally defined roles, responsibilities, and household dynamics between male and female caregivers that could also play a role in getting children vaccinated. In many contexts, women are the primary caregivers of children and thus responsible for seeking their child's vaccination (6). Women are also often the frontline health worker in many settings and thus responsible for providing vaccination. There is a need to understand how factors unique to female caregivers affect childhood immunization and examine the interrelationships of these factors across the individual, household, community, and health system levels. The socio-ecological model (SEM) can help develop multi-level programs to improve MCV2 coverage. This study aimed to explore barriers to MCV2 and childhood immunization specific to female caregiver's face in Uganda.

These barriers specific to female caregivers may operate at multiple levels (7), at multiple levels, therefore it is important to identify key barriers at different levels. At the: individual level, female caregivers face time constraints, financial limitations, low prioritization of immunization, low health literacy, and low acceptability of health services. At the household level, interactions between male and female caregivers affect decision-making and access to household resources, with women often having reduced control over health-related decisions. At the community level: cultural factors like ethnicity and religion, and women's limited mobility and participation in community decision-making influence vaccination uptake, Health system level: Barriers include poor caregiver-provider interactions, lack of access to healthcare facilities due to inconvenient times or distance, and inadequate healthcare service quality, Policy level: Issues such as governance, stakeholder engagement, and the policies (8), laws, and regulations impacting immunization services.

This paper aims to describe female caregiver-specific barriers to MCV2 and childhood immunization specific using a rapid community assessment (RCA) conducted to inform MCV2 roll out in Uganda.

## 2 Methods

### 2.1 Study design

In September 2022, we conducted a rapid community assessment (RCA) (9) in 18 districts in Uganda, using qualitative methods such as focus group discussions (FGDs) with caregivers and key informant interviews (KIIs) with health managers and community leaders to gather insights into the community's perceptions of the newly introduced vaccination program. The qualitative assessment was done prior to the planned national implementation of the MCV2 vaccine roll out in Uganda.

### 2.2 Setting

The public healthcare facilities and communities for the RCA were selected based on data from the Uganda National Immunization survey conducted in 2017 under the Uganda National Expanded Programme on Immunization (UNEPI).

### 2.3 Sampling

Eighteen districts were selected in consultation with UNEPI partners based on measles vaccine coverage and recent outbreaks. Eight districts had high measles vaccine coverage (≥80%), eight had low coverage (<50%), and two had reported measles outbreaks in 2020 (Supplementary Material 1). Districts with outbreaks, as defined by the National Guideline on Measles Surveillance and Outbreak Management, had at least five laboratory-confirmed measles cases in 1 month.

For KIIs, we purposively selected 17 KIIs health managers, i.e., Assistant District Health Officials assigned to each district and 18 community leaders [e.g., local chairpersons, Village Health Team (VHT) coordinators, and women representatives], one in each district. One FGD was conducted in each district, with 6–11 participants per group, purposively selected from caregivers of children under 5 years. The district health officials and VHTs helped identify and recruit caregivers from the community. Written informed consent was obtained for all participants.

### 2.4 Data collection

Data collection involved inviting KII and FGD participants either in person or by phone to participate in discussions held in private locations, such as district offices or local chairpersons' offices. FGDs complemented the KIIs by gathering insights on perceptions about of vaccination (10). Tailored discussion and interview guides with open-ended questions were used for different participant groups. KIIs with health managers focused on health system factors and MCV2 rollout preparations, while KIIs with community leaders addressed community norms, vaccine acceptability, and strategies to address male or female-related barriers. Caregivers and community leaders were asked about their experiences and perspectives on vaccination (Supplementary Material 2–4).

We conducted data collection in one of ten languages (i.e., English, Luganda, Runyakitara, Lugbara, Lugisu, Lugungu, Atesot, Kumam Karamajong, and Sebei) depending on the geographical area and participant preference. The FGD guide was interpreted in real-time by an interviewer familiar with the local language and a fluent third-party interpreter, when necessary. The external interpreter assigned by the ADHO, understood the local language and was briefed on the study's purpose and confidentiality requirements. Participants provided written informed consent, with thumbprints used for those who couldn't write.

KIIs lasted approximately 25–60 min and FGDs lasted approximately 40–90 min. Interviews were audio-recorded with consent, transcribed verbatim and, where needed, translated into English for data analysis. Data collection took place for over 2 weeks, with on-going rapid analysis of debrief forms. The debrief form consisted of key themes and issues that emerged from the discussions. A separate debrief form was completed for each discussion jointly by the facilitator and note-taker.

### 2.5 Data analysis

Data analysis involved two phases. Phase 1 was a rapid synthesis based on debrief forms and field notes from KIIs and FGDs. Three coders (AT, NR, and SK) independently reviewed debriefing notes, identified recurring themes, and reached consensus on key barriers to childhood immunization and MCV2 uptake. Phase 2 involved a more in-depth analysis of transcribed and translated interviews, using both deductive and inductive coding methods to identify emerging themes that we mapped onto the socio-ecological model (8, 11). Illustrative quotations were selected to support the findings, which were presented following the COREQ checklist for qualitative research (12).

## 3 Results

### 3.1 Participant characteristics

Of the 35 KIIs Informant Interviews (KIIs) conducted with health managers 59% were female, while 67% of the 18 community leaders were female. For the 18 Focus Group Discussions (FGDs) were held, most participants (121, 94%) were female.

### 3.2 Factors affecting immunization

Individual, household, community, and health system factors influenced decisions on childhood vaccination, particularly for the second dose of the measles-containing vaccine (MCV2). Specifically, among men and women caregivers. Male or female barriers were highlighted through the socio-ecological model.

#### 3.2.1 Individual-level factors affecting immunization

##### 3.2.1.1 Paucity of time for female caregivers

Individual-level factors affecting immunization role differentiation with women often taking on the primary caregiving responsibilities which can cause time paucity of mothers to take their children for timely vaccination. For example, with the Uganda context in the 18 districts, women's multiple tasks, such as gardening during rainy season, livestock rearing, cooking, washing, and ironing clothes that they were primarily responsible for and thus affected the time that could be devoted to taking the child for vaccination.

“[Taking the child for vaccination] may affect my daily activities. Sometimes, when they [neighbors] hand me the baby to look after yet I have not planned for it, it disrupts my agenda. Sometimes, I may have to feed my cow and when I fail to cut some grass to feed it, it gets hungry and all that is a result of an announcement of abrupt outreach in the community to vaccinate the child.”

 – Woman caregiver, FGD, high vaccination coverage district

##### 3.2.1.2 Low decision-making power

Some female caregivers reported having less decision-making power in the household, often relying on their husband's approval for vaccination, which contributed to delays or refusals if the father was unsupportive of vaccinations. Financial constraints also play a role, as women often depend on their husbands for money to cover transportation and other vaccination-related costs, that could further hinder access to MCV2 vaccination.

“Very few of us go to the health centre. Out of 15 people that need vaccination, only 5 or 7 will reach the health centre. The rest will fail due to financial constraints. I can tell my husband that the child needs to be vaccinated and he replies: ‘… I do not have that money today.’ If I do not my personal money, I simply dodge vaccination.”

 – Woman caregiver, FGD, low vaccination coverage district

#### 3.2.2 Household-level factors affecting immunization

##### 3.2.2.1 Intrahousehold disagreement on child vaccination

Conflict was reported as a factor relating to childhood immunization by caregivers and community leaders. Most women reported that vaccination was a common reason for their children to cry due to discomfort from the painful injection and side effects from immunization. This sometimes led to the husbands getting aggressive toward the woman.

“Sometimes, the child’s father says, “My child should not be vaccinated”—because…child may get worse…When you tell him (father) that the child fell sick, he will tell you, ‘Why did you take the child to the hospital to be immunized?’ When the child cries, he will say, ‘I do not want to hear the child making noise for me all night. Do not take the child for immunization again without my permission.’ When you oppose him, it only spikes conflict between the both of you. I have to handle my spouse with care and come to a mutual understanding. If he prohibits me from taking the child, I should not oppose him. If he allows, then I can take the child.”

 – Woman caregiver, FGD, recent measles outbreak district.

“Some women caregivers may resist taking their children for another vaccination because of the painful after effects of the injections causes. It causes the child to cry a lot that angers the man in the house.”

 – Community leader, KII, high vaccination coverage district

##### 3.2.2.2 Low male involvement in vaccination

Participants generally reported low male support for childhood vaccination. A few participants did suggest some men are involved in a child's vaccination, but others were not supportive due to lack of time and critical need to focus on earning a living for the family.

“Maybe male involvement [in childhood vaccination] is very low. You find some families, in most cases here in Buganda, it is the man to decide. Most men are very rigid when it comes to health issues. Remember, we mainly have only women at health centres. When I talk to a woman, and they give feedback to the husband, he will tell her, ‘Do not waste time.’ They do not have time to come for these health talks [patient health education in the facility]. Even when you reach them in their communities, they already do not have time. That is why for that kind of family where men decide and they take the decision not to vaccinate the child, then it means they will not vaccinate that child.”

 – Health manager, KII, high vaccination coverage district

#### 3.2.3 Health facility level factors affecting immunization

HCW gave preference to male caregivers when attending for child vaccination as compared to female caregivers. Participants reported the practice of HCW prioritized attending male caregivers who brought their child for vaccination over female caregivers. While this was seen as a facilitator to engage more males in health-seeking behaviour, for mothers, it may also be seen as an action that could undervalue mother's time as compared to father's time, and potential cause additional challenges related to time paucity and delayed vaccinations.

“There are some men who bring their children in person because we have a strategy that if a man brings his own child, they are given priority… men cannot breastfeed babies if they are delayed at facilities. Sometimes I find men in queues for child vaccination, we also try to give them some health talks to go and teach fellow men.”

 – Health manager, KII, high vaccination coverage district

Caregivers reported women also faced stigma at health facilities, especially those with closely spaced children, which impacted their willingness to seek timely vaccinations.

“*What usually hinders women from taking their children for vaccination on time, you find a woman has a child of six months and she is three months pregnant. Now she becomes lazy to take the child for vaccinating; she thinks to herself, when I get there, they will say ‘You are pregnant, yet the child is young, and you have brought it to hospital.’ So that is what she fears. So, she says, ‘When I give birth, I will eventually take both.’ But she ends up taking the younger one because she lacks money for transport for two children*.”

 – Woman caregiver, FGD, high vaccination coverage district

“Some mothers may fear going to the health facility knowing that during immunization, health workers ask for father’s name and yet they want to keep it a secret.”

 – Woman caregiver, FGD, high vaccination coverage district

### 3.3 Strategies to address female caregiver-specific barriers to vaccination

Participants suggested several strategies (Box 1) to potentially address barriers that female caregivers faced:

Box 1 **Recommendations from participants to address challenges to second dose of measles-containing vaccine (MCV2) using approaches that address barriers facing female caregivers.**

• Health care workers should strengthen couples counselling in communities and health facilities or establish strategies on targeting families rather than only mothers, so that fathers and others family members are also aware of MCV2.
• Healthcare workers should attend to mothers and other caregivers who have brought children for vaccination in a respectful and timely manner.
• VHTs should find mothers and fathers in their homes or gardens to inform about dates for MCV2 vaccination and sensitize families to take personal responsibility for childhood vaccination.
• A peer-to-peer approach could be utilized that builds on trusted relationships and social norms developed in mother's groups.
• Community leaders should be sensitized about MCV2, so that they can educate men to support women during childhood vaccination.

#### 3.3.1 Family-based approaches

Participants suggested sensitization of families to take joint responsibility for vaccination and educate fathers on the importance of supporting childhood immunization. This was thought to potentially also support joint decision-making and fostering a more supportive environment for vaccination at the household level.

“We would like to have an education session for men, to help them to support their wives…the man should know that the child is for both parents…It means if the mother gets sick and the man is not supportive, there will be no one to take the child for routine immunization.”

 – Village Health Team coordinator, KII, recent measles outbreak district

Participants suggested counseling efforts to target families with negative attitudes toward MCV2 vaccination, with VHTs providing information and collaborating with local authorities to address any vaccination hesitancy or concerns. Participants suggested that VHTs should find mothers and fathers in their homes to inform about dates for MCV2 vaccination and sensitize families to take personal responsibility for childhood vaccination.

“Counselling of some families that could be having a negative attitude about immunization. Such families should also be reported to the local council chairperson who could visit these homes to get to know why such families don’t immunize their children. Through such talk, those members can be convinced and end up taking their children for immunization.”

 – Woman caregiver, FGD, low measles coverage district

“The health worker must counsel the two parents concerning the importance of the vaccine drug which they are given. The parents must take care of the children willingly. When an appointment is scheduled, they both must turn up as the parents. Let the mother not come alone so that the father will also listen to the advice that is usually given to the mother alone. They must join hands in bring[ing] up the child, so that it helps…”

 – Woman caregiver, FGD, high vaccination coverage district.

#### 3.3.2 Community approaches

Some female caregivers and VHT leaders suggested utilization of peer-to-peer approaches and involvement of community leaders to promote MCV2 awareness and encourage male support.

“This information that you have given us (about MCV2) will be carried as gospel, so we [peers] will inform our colleagues who have not been around. By the time you return, we [peers] will have some knowledge and so you will find us prepared. It would be good if you return, it will become a gospel and we will gather our colleagues so that we are many.”

 – Woman caregiver, FGD, low vaccination coverage district

#### 3.3.3 Health system approaches

Participants said health managers should ensure that healthcare workers attend to caregivers in a timely and respectful manner to minimize delays and reduce stigma, treat caregivers with respect to ultimately help foster trust.

“What is required of them is to attend to us in a timely manner so that we can go back home early. They should attend to us in a timely manner—because some doctors come late, and they begin moving about yet you came earlier and having been for them. The service we want from them is for them to attend to us very fast so that we can go back home”

 – Woman caregiver, recent measles outbreak, district.

## 4 Discussion

Our findings suggest that female caregivers experience several unique barriers that may hinder childhood vaccination within communities in Uganda. Using a socio-ecological model, we illustrated the multilevel interconnectedness of these barriers to childhood vaccination, and how they may also shape demand for and access to the MCV2 introduction being implemented nationally for children in the second year of life.

At the individual level, women caregivers reported having less decision-making power than men, being financially dependent on the child's father, and having a high workload and paucity of time, all of which hindered their ability to take children for timely vaccination (13, 14). Various studies highlight that with increased autonomy and decision-making capability, women caregivers are more likely to immunize their children (11, 15). While in instances of low decision-making power, many women may not feel free to take a sick child to the doctor without the approval of their husband or parent-in-law (16, 17).

Male involvement is often lacking in child immunization, but often men play a significant role in health-related decisions. Women are more often the primary caregiver within the family, men also shape women caregivers' seeking of health services for the children within a household. Women, especially in resource-constrained or emergency settings, tend to have poorer access to, and control over, critical resources such as time, money, and transportation (18). Given the crucial role African men play in family decisions, their support and involvement in the household health care are essential for healthy maternal and child welfare (19). Meaningful men's engagement in childhood vaccination can boost support for women that enhances childhood vaccination. Integrating women and men as active partners in health programs could be a useful strategy to transform inequalities among male or female.

At the household level, we found that intrahousehold disagreement on vaccinations also negatively impacted immunization outcomes mainly due to husband's aggression if a child is upset or crying due to immunization side effects (20). Some prior literature highlights children of mothers experiencing violence or aggression in the household are less likely to be fully immunized than children of women not experiencing it. Women abstain from seeking healthcare for children to avoid more physical violence and abuse (21, 22).

Preferential treatment of male caregivers was reported at the health facility level: Male caregivers were treated more favorably at health facilities, which could be a result of bias or other social factors, that prioritize male involvement in caregiving. Study suggests unique factors alongside other dynamics, which may create different opportunities and challenges for male and female caregivers when it comes to healthcare access and decision-making regarding immunization. Responsive activities, including male engagement and collaboration with various stakeholders, are crucial to improving vaccination coverage.

Peer-to-peer approaches, caregiver-specific programs, and continued research are needed to address these barriers and enhance immunization uptake in Uganda. Participants suggested the peer-to-peer approach as it leverages trusted relationships to address social norms and promote support, ultimately boosting vaccine uptake. MCV2 demand-generating programs should be specific, tailored to address the specific needs of female and male caregivers. Ideally, these programs could also aim to improve unequal household dynamics, societal norms, roles, and practices (23).

Future research should continue to examine gender-related barriers after the implementation of MCV2 among female caregivers in Uganda to have a greater understanding of the role of mothers and fathers in second year-of-life vaccination platform. These barriers should also be examined and addressed in varied settings where challenges to adequate immunization uptake persist, including among hard-to-reach populations, zero-dose communities, and in other settings where new vaccines or doses are introduced.

## 5 Strengths

This assessment is one of the first in Uganda to study barriers specific to female caregivers using a rapid community assessment approach within the context of introduction of MCV2 introduction. Results from the rapid analysis were shared to inform UNEPI about the local needs and priorities in the selected districts for MCV2 roll out and programming for future vaccination. The results helped identify the need to focus on awareness creation, social mobilization, and community engagement. Assessing these barriers from multiple respondents' perspectives provided additional validation of common issues which female caregivers may face when accessing immunization. The mapping of the barriers on the various socio-ecological levels provided evidence for developing caregiver-level and health system-level interventions that can address these issues.

## 6 Limitations

The limitations of this assessment include a risk of social desirability bias and potential under reporting of gender-related barriers among participants who might have been hesitant to share detailed experiences. All participants were at least 18 years old, and the majority were females, since most caregivers are female; future research could purposively involve caregivers who are men and emancipated minors to gather more perspectives. Given the qualitative nature of this assessment, we could not make inferences to potential vaccine uptake or coverage.

## 7 Conclusions

Understanding barriers to immunization goes beyond concerns of coverage disparities between girls and boys (24), it includes multi-level factors that shape demand, supply, and uptake of vaccination, particularly in countries with strong traditional roles among males and females. In Uganda, role differentiation among caregivers' shape childhood vaccination practices and may exacerbate difficulties in accessing measles-containing vaccines for children in their second year of life. Health programs should incorporate programs that create opportunities for male and female caregivers to be involved childhood immunization by implementing targeted interventions in education, community awareness, and policy reforms. Specific interventions should be implemented along with interventions that address barriers such as education and income inequalities. From our experience, these inequalities education, income have underlying gender disparities.

## Data Availability

The original contributions presented in the study are included in the article/Supplementary Material, further inquiries can be directed to the corresponding author.
