# Supplementary material for: “Vaccinating a child is upon the woman”: implications for improving uptake for the recently introduced second dose of measles-containing vaccine based on a rapid community assessment in Uganda
Source: Front Glob Womens Health. 2025 Apr 11;6:1441242. doi: 10.3389/fgwh.2025.1441242 (PMC12021830; doi:10.3389/fgwh.2025.1441242)
Supplement: Supplementary file 1 [file Table1.docx]

**Additional file 5: KNOWLEDGE AND PERCEPTIONS OF SECOND DOSE OF MEASLES VACCINES AMONG CAREGIVERS AND HEALTH CARE WORKERS IN UGANDA**

**Code book-1/07/2023.**

Nodes

| Name | Description |
| --- | --- |
| **GENERAL KNOWLEDGE AND PERCEPTIONS ABOUT ROUTINE IMMUNIZATION** | **Description about perception about benefits of routine vaccination, generally positive social norms, negative perceptions, and Perception of child health services accessed by child and caregivers** |
| Barriers to routine vaccination |  |
| Facilitators of routine vaccinations |  |
| **GENERAL KNOWLEDGE AND PERCEPTIONS OF MEASLES DISEASE AND VACCINATION** | **What participants described as perceptions about measles disease, signs and symptoms including management of measles disease among children, general positive and negative attitudes toward measles vaccination as well as discussion about how there may or may not be an outbreak.** |
| Challenges experienced during previous measles vaccination |  |
| General positive attitudes toward measles vaccination |  |
| Information share by HCWs about measles |  |
| Management of measles disease among children |  |
| Measles transmission, diagnosis |  |
| Signs and symptoms, |  |
| Source of measles vaccine |  |
| **KNOWLEDGE AND PERCEPTIONS OF 2YL VACCINATION** | **Description of perceptions and knowledge about 2YL Vaccination including benefits to child, myths and misconceptions among care givers, willingness and readiness to accept MR2, plan for roll out.** |
| Acceptability of MR 2 |  |
| Awareness about MR 2 rolls out |  |
| Lessons learnt from previous vaccinations to inform MR.2 roll out |  |
| Mixed feelings about MR 2 introduction |  |
| Positive Perceptions about MR2 |  |
| Wait to take their children to get MR2 and see approach to vaccination |  |
| **POTENTIAL BARRIERS OF MR2 ROLL OUT** | **What participants described as potential barriers to MR2 Roll out at family, individual, community, facility, government or MOH levels.** |
| Caregiver barriers |  |
| Community issues |  |
| Gender barriers to immunization |  |
| Health systems issues |  |
| Infrastructure issues |  |
| **SUGGESTED STRATEGIES FOR KNOWLEDGE AND ACCESS OF MR 2** | **Potential suggestions of MR2 roll out at Individual, facility and community level.** |
| Community-based approaches |  |
| Family-based approach |  |
| Government |  |
| Health system approaches |  |
| Mobilizing and engaging with religious leaders. |  |
| Peer-based approaches |  |
| Personal responsibility |  |
| School approach |  |
| Use of information, communication, education (IEC) materials, posters, and megaphones |  |
