# Supplementary material for: “Vaccinating a child is upon the woman”: implications for improving uptake for the recently introduced second dose of measles-containing vaccine based on a rapid community assessment in Uganda
Source: Front Glob Womens Health. 2025 Apr 11;6:1441242. doi: 10.3389/fgwh.2025.1441242 (PMC12021830; doi:10.3389/fgwh.2025.1441242)
Supplement: Supplementary file 2 [file Table2.docx]

**Additional file 4: Key Informant topic guide for Health Managers (V1.1-21.7.22)**

Participant ID # ___________ Interviewer_________ Audio file #: ____________ Date____

Participant type: ADHO- MCH other (Specify)______________

**Introduction**

| Welcome and thank you for accepting to participate in this interview. My name is …[NAME], I will be conducting this interview. Infectious Diseases Institute in collaboration with AFENET, MOH, NIPH, UNEPI and CDC launched out to Identify knowledge and perceptions of second dose of measles vaccines among caregivers and health care workers in Uganda.  As you may know, the second dose of measles vaccine (MR2) is needed to offer full immunity to children against this disease. UNEPI is planning to nationally introduce this second dose soon. We would like to hear from you to gain insights on the roll out of this vaccine, any planning that is being done in this district, and strategies for MR2 introduction. You are the expert in your experience, and your thoughts and opinions are greatly valued and appreciated. We shall be asking you about your opinions about childhood vaccination activities, measles campaign, introduction of MR2 and suggestions for a successful MR2 roll out.    Your participation is voluntary, anonymous with no names recorded and your individual level information not shared.  You can choose to stop participating at any time. We expect not to spend more than 45 minutes during this discussion.  We will take notes during the interview and will audio record, when possible, to help capture your comments accurately and complete our notes. We will destroy the recordings after we make the notes. You can choose not to respond to a question at any time and your participation in this assessment is voluntary, and you can leave at any time.  Today’s interview should take about 45 minutes.  Before we start, do you have any questions about today’s interview? |
| --- |

1. To begin, can you describe your role in supporting immunization activities in this district? Probe: can you describe your role in supporting immunization activities in this district or health facility.
2. Please tell me what you know about the proposed roll out of MR2.
3. What are your thoughts on the upcoming introduction of MR2?
   1. Probe: Do you think a second dose is necessary? How?
   2. Probe: Timing of introduction
4. How are you/ how is the district planning to introduce MR2?
   1. Probe: Are there any training plans for the HCW and supervisors for MR2 introduction?
   2. What kind of training? Please elaborate on that
   3. Probe: Updating immunization documentation: child health cards, registers etc.
   4. Probe: How are you going to raise awareness about MR2.?
      1. What type of messaging is being planned?
      2. Channels to be used
      3. Frequency of social mobilization
   5. Probe: How is the initial service delivery of MR2 being planned (fixed session, outreach sessions, ICHDs)?
   6. Probe: How will this introduction be integrated into routine schedule after initial introduction of the vaccine?
      1. Any guidelines for updated processes for documentation for child health cards, immunization registers
   7. Probe: How will the initial introduction be evaluated?
   8. Probe: Is there any additional planning being done for the introduction?
      1. Example: human resources, financial planning
5. What do you think are some potential facilitators in this district that can help with MR2 introduction?
   1. Community level factors
   2. Health system factors
   3. Any lessons learned from any other recent vaccine introduction?
6. What do you think are some potential barriers in this district that can affect MR2 introduction?
   1. Community level factors
      1. Community perceptions about immunization services
      2. Current ease of getting immunization services (household and logistical factors)
   2. Health system factors
      1. Additional work burden, responsibilities
      2. What additional skills/ resources would HCW need?
   3. Probe about how the challenges may be mitigated
   4. based on your experiences, what are your suggestions for strategies…”
   5. What partners have been the most committed to engaging in messaging activities?
7. “Can you describe any experience you have with measles campaigns? What was easy or challenging about the activity?”
   1. Probe: Planning for the campaign
   2. Probe: Communication for the campaign
   3. Probe: Evaluation of campaign activities
   4. Probe: What types of campaigns (messages, medium) have seemed to have the biggest impact on the community’s practices?
8. Do you have any suggestions for strategies for a successful MR2 rollout?

Probe: Ideas for integration of MR2 delivery with on-going health services for mother and child in the second year of life.

Is there anything else you expected me to ask about MR2 rollout that I did not ask?

Thank you
