# Supplementary material for: “Vaccinating a child is upon the woman”: implications for improving uptake for the recently introduced second dose of measles-containing vaccine based on a rapid community assessment in Uganda
Source: Front Glob Womens Health. 2025 Apr 11;6:1441242. doi: 10.3389/fgwh.2025.1441242 (PMC12021830; doi:10.3389/fgwh.2025.1441242)
Supplement: Supplementary file 3 [file Table3.docx]

**Additional file1: Table showing UNEPI list of low and high measles immunization coverage districts where RCA was conducted.**

|  | **Low Measles Immunization Coverage Districts <50%.** | |  | **High measles immunization coverage >80%%.** |
| --- | --- | --- | --- | --- |
| **1** | **Kasese: (Western)**  Muhokya Subcounty (Nyamirami HC IV), Lubiriha Town Council (Bwera Hospital), Hima Town Council (Hima HC III) |  | **1** | **Kassanda (Central)**  Makokoto Subcounty (Bbira HC II), Bukuya Subcounty (Bukuya HC III), Kassanda Town Council (Kassanda HC IV), Kiganda Subcounty (Kiganda HC IV). |
| **2** | **Moyo: (West Nile)**  Metu Subcounty (Abeso HC II), Moyo Town Council (Besia HC III, Moyo Hospital), Moyo sub county (Moyo Mission HC IV) |  | **2** | **Namisindwa (Eastern)**  Bubutu Subcounty (Bubutu HC III), Bukhaweka Subcounty (Bukhaweka HC II), Magale Town Council (Magale (UCMB) HC IV), Magale Subcounty (Magale Hans HC III), |
| **3** | **Rwampara: (Western)**  Bugamba Subcounty (Bugamba HC IV), Rugando Subcounty (Kinoni HC IV), Ndeija Subcounty (Kongoro HC II), Mwizi Subcounty (Mwizi HC III) |  | **3** | **Manafwa (Eatern)**  Manafwa Subcounty (Bubulo HC IV), Bugobero Subcounty (Bugobero HC IV), Butiru Subcounty (Butiru Dispensary HC II, Butiru HC III). |
| 4 | **Bullisa: (Western)**  Biiso Subcounty (Biiso HC III),  Buliisa Subcounty (Buliisa Hospital), Buliisa Subcounty (Buliisa Hospital) |  | **4** | **Obongi (West Nile)**  Aliba Subcounty (Aliba HC III), Itula Subcounty (Belameling HC II, Palorinya HC III), Obongi Town Council (Obongi HC IV) |
| **5** | **Moroto: (Karamoja)**  Nadunget Subcounty (Acherer HC II, Loputuk HC III, Nadunget HC IIV), Northern Division (Moroto MC) (Moroto Regional Referral Hospital). |  | 5 | **Bududa(Eastern)**  Bududa Town Council (Bududa Hospital), Bukigai Subcounty (Bukigai HC III), Bumayoka Subcounty (Bufuma HC III), Nalwanza Subcounty (Bumusi HC II), Bushiyi Subcounty (Bushiyi HC III). |
| 6 | **Nakasongola: (central)**  Kalongo Subcounty (Bamugolodde HC III), Lwampanga Subcounty (Kikoiro HC II), Nakasongola Town Council (Nakasongola HC IV), Lwampanga Subcounty (Nakasongola Military Hospital) |  | 6 | **Adjumani:** **(West Nile)**  Adjumani Town Council (Adjumani Hospital, Adjumani Mission HC III), Ciforo Subcounty (Agojo HC II), Itirikwa Subcounty (Mungula HC IV). |
| 7 | **Kalaki(Eastern)**  Kalaki Subcounty (Kalaki HC III), Kakure Subcounty (Kakure HC III), Otuboi Subcounty (Lwala Hospital, Maddona HC II) |  | 7 | **Bunyangabu:** **(Western)**  Kabonero Subcounty (Kabonero HC III),  Kibiito Town Council (Kibiito HC IV), Kisomoro Subcounty (Kisomoro HC III) |
| 8 | **Serere: (Eastern)**  Pingire Subcounty (Aarapoo HC III), Bugondo Subcounty (Apapai HC IV), Olio Subcounty (Miria HC II), Serere Town Council (Serere HC IV) |  | 8 | **Sironko (Eastern)**  Budadiri Town Council (Budadiri HC IV), Bugitimwa Subcounty (Bugitimwa Govt HC III), Buwasa Subcounty (Buwasa HC IV), Nalusaala Subcounty. (Buyaya HC II) |
|  |  |  |  |  |
|  | Districts with recent measles outbreak | | | |
| 1 | **Kween: (Eastern)**  Kaproron Town Council (Kaproron HC IV), Kaptum Subcounty (Kaptum HC III) Binyiny Town Council (Binyiny HC III), Benet Subcounty (Likil HC II). | | | |
| 2 | **Nakaseke: (Central)**  Kasangombe Subcounty (Bidabuja HC III), Semuto Subcounty (Kalege HC II), Ngoma Town Council (Ngoma HC IV), Nakaseke Town Council (Nakaseke Hospital). | | | |
|  |  | | | |
