# Supplementary material for: “Vaccinating a child is upon the woman”: implications for improving uptake for the recently introduced second dose of measles-containing vaccine based on a rapid community assessment in Uganda
Source: Front Glob Womens Health. 2025 Apr 11;6:1441242. doi: 10.3389/fgwh.2025.1441242 (PMC12021830; doi:10.3389/fgwh.2025.1441242)
Supplement: Supplementary file 4 [file Table4.docx]

**Additional file 2: FDG DISCUSSION GUIDE FOR CAREGIVERS WITH CHILDREN AGED 12-59 MONTHS (V1.1-21.7.22)**

FGD ID #: ______________ Moderator Initials: __________Note-taker Initials: __________

Participant group_____________ # of participants_________ Audio file # _________________

Community: _____________________ Date of FGD ________________________

**Introduction**

| Welcome and thank you for accepting to participate in this focus group discussion. My name is …[NAME], I will be facilitating today’s focus group discussion, and … [NAME] will be taking Notes. Infectious Diseases Institute in collaboration with AFENET, MOH, NIPH, UNEPI and CDC launched out to Identify knowledge and perceptions of second dose of measles vaccines among caregivers and health care workers in Uganda.  The purpose of this focus group is to help us learn about your opinions about vaccinations for children, second dose of measles vaccination a year old/ second year of life, potential limitation/ barriers with taking children to health facilities for measles vaccine and suggest strategies to promote measles vaccine uptake. We shall use the information you share with us today to improve uptake of measles vaccination in your community.  We would like to hear your honest opinions about the topics we discuss. There are no right or wrong answers to any of the questions we ask today. You are the expert on your experience, and your thoughts and opinions are greatly valued and appreciated. We encourage you to speak openly and honestly about your opinions and experiences. You can choose not to respond to a question at any time and your participation in this assessment is completely voluntary, and you can leave at any time. We will be audio recording this session and also taking notes. The recordings and notes will help us to summarize today’s discussion. None of the comments you make during today’s discussion will be linked with your name in any way and all information from this discussion will be summarized anonymously.  Today’s discussion should take about 45- 90 minutes.  Before we start, do you have any questions about today’s discussion? |
| --- |

1. What do you think about vaccinations for children?
2. Probe: Individual feelings about child being vaccinated
3. Probe: What do other families in your community think about vaccinations for children?
4. Probe: Where are children taken for vaccination?
5. Why would you bring a child to a health facility after he or she is one year old? Explain response.
6. Probe: What services did you seek at that time?
7. Probe: How often do you go to the health facility in the second year?
8. Probe: Do you face any issues for these visits in the second year?
9. What do you know about measles?
10. Probe: Symptoms of the diseases
11. Probe: Who is at risk and how severe is the disease?
12. Probe: What was experience getting the first dose of measles vaccine (given at 9 months)?
13. Probe about the nature and quality of services/information received about first dose of measles vaccine (given at 9 months)

Probe about relationship with health workers during first dose of measles vaccine (given at 9 months)

1. Probe: Any prior experience with measles in this community?

Some diseases also require additional doses of vaccines after he or she is one year old to offer full protection. If the immunization schedule is updated where a child would also need to get vaccines in the second year, what are your thoughts on taking your child for additional vaccine doses to the health facility after he or she is one year old?

1. How would you feel if vaccines were provided after a child turns one?
2. What potential issues do you expect for taking your child to the health facility after the child is one year old/ older than one year
   1. Probe on access issues
   2. Probe: Household decision-making process to get child vaccinated for additional doses
      1. Who will make the decision? How are decisions about child vaccination made in the family for additional doses (what role do men or women play).
   3. Probe: How will taking the child to the health facility affect your day-to-day activities/ schedule/ commitments, priorities?
      1. Household chores
      2. Work (income generating activities)
      3. Time constraints
      4. Caring for other family members
   4. Probe: How do you think your community members might support or resist immunization after your child is one year old? in your child’s second year of life?
      1. Who will support and who will resist?
3. If a second dose of measles vaccine is introduced in near future, what are some of the things that would make it easier for caregivers to get their child vaccinated? Probe: what do you think would make caregivers accept to get child vaccinated for second dose of measles vaccine?
   1. Probe: Information about second dose of measles vaccine for caregivers and community
      1. How would you like to get this information? From whom? Where?
   2. Probe: Anything at the household level?
   3. Probe: What will help in accessing the health facility?
   4. Probe: how the sociocultural barriers identified may be addressed?
   5. Probe: How can HCW help support caregivers for getting their child vaccinated?
      1. For awareness, understanding, reminders?
4. What other health services would you want for yourself and/or child when you take your child for immunization in the second year?
5. What strategies can be implemented in the community for creating knowledge and awareness on second dose of measles vaccine?
   1. Who in the community should be engaged to support introducing the second dose of the vaccine?
   2. What activities should be conducted to engage with the community of vaccination?
6. What strategies can be implemented in the community to increase access to second dose of measles vaccine?

Is there anything else you expected me to ask about MR2 rollout that I did not ask?

We have come to the end of our discussion. Thank you very much for your time.
