# Supplementary material for: “Vaccinating a child is upon the woman”: implications for improving uptake for the recently introduced second dose of measles-containing vaccine based on a rapid community assessment in Uganda
Source: Front Glob Womens Health. 2025 Apr 11;6:1441242. doi: 10.3389/fgwh.2025.1441242 (PMC12021830; doi:10.3389/fgwh.2025.1441242)
Supplement: Supplementary file 5 [file Table5.docx]

**Additional file 3: KEY INFORMANT TOPIC GUIDE FOR KEY COMMUNITY REPRESENTATIVES (V1.1-21.7.22)**

Participant ID # ___________ Interviewer_________ Audio file #: ____________ Date____

Participant type: village health team leader women’s group leader
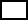
 Religious Leader
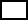


LC1 chairman other (Specify)__________

**Introduction**

| Welcome and thank you for accepting to participate in this interview. My name is …[NAME], I will be conducting this interview. Infectious Diseases Institute in collaboration with AFENET, MOH, NIPH, UNEPI and CDC launched out to Identify knowledge and perceptions of second dose of measles vaccines among caregivers and health care workers in Uganda.  We would like to hear from you to gain insights on the roll out of this vaccine, any planning that is being done in this district, and strategies for MR2 introduction. You are the expert in your experience, and your thoughts and opinions are greatly valued and appreciated. We shall be asking you about your opinions regarding vaccinations for children in the community, measles vaccination activities and implementation of measles in second year.  We would like to hear your honest opinions about the topics we discuss. There are no right or wrong answers to any of the questions we ask today. You are the expert on your experience, and your thoughts and opinions are greatly valued and appreciated. We encourage you to speak openly and honestly about your opinions and experiences. You can choose not to respond to a question at any time and your participation in this assessment is voluntary, and you can leave at any time. We will be audio recording this session and also taking notes. The recordings and notes will help us to summarize today’s discussion. None of the comments you make during today’s discussion will be linked with your name in any way and all information from this discussion will be summarized anonymously.  Today’s discussion should take about 45 minutes.  Before we start, do you have any questions about today’s interview? |
| --- |

We will start our discussion with vaccinations for children in your community.

1. What do you think about vaccinations for children in your community?
   1. Probe: What challenges does the community have in getting children vaccinated
   2. Probe: How do people in your community usually talk/ perceive vaccinations for children or vaccination services at health facility or outreach in your community?
   3. Probe: what are the key barriers in your community for childhood vaccines?
2. What do you know about measles?
   1. Probe: Symptoms of the diseases
   2. Probe: Who is at risk and how severe is the disease?
   3. Probe: What is your prior experience with measles in this community?
3. Do caregivers in your community go to the health facility for any child health services after he or she is one year old?
   1. Probe: If yes, which services? Do you know what age do they usually visit the health facility for these services?
4. If children require one or more vaccines after their first year of life, how would you feel about it as a community leader?
   1. Probe: what would your role be as a community leader be around these vaccines?
5. What potential issues or challenges might the community face if caregivers need to take their children to vaccinate after he or she is one year old?
   1. Probe: Any issues related access?
   2. Probe: Any household issues?
   3. Probe: Any issues at the health facility?
6. What strategies can community leaders use to make sure children go for vaccines after he or she is one year old in your community?
   1. Probe: How can community engagement be conducted?
   2. Probe: How can community leaders work with community health workers or HCW?
   3. Probe: Who else in the community should be engaged to support introducing the second dose of the vaccine?
7. Is there anything else about this topic that you would like me to know?
8. Is there anyone else who you think it would be important for me to speak with for additional information on this topic?

Is there anything else you expected me to ask about MR2 rollout that I did not ask?

We have come to the end of our discussion. Thank you very much for your time.
